# Agile Thinking Slays the Chimaera

**DOI:** 10.3201/eid2809.AC2809

**Published:** 2022-09

**Authors:** Byron Breedlove

**Affiliations:** Centers for Disease Control and Prevention, Atlanta, Georgia, USA

**Keywords:** art science connection, emerging infectious diseases, art and medicine, about the cover, high consequence pathogens, viruses, virus diseases, monkeypox, hemorrhagic fever diseases, Middle East respiratory syndrome, rabies, Nipah virus infection, Ebola virus disease, Marburg hemorrhagic fever, Crimean-Congo hemorrhagic fever, Rift Valley fever, chimaera, Agile Thinking Slays the Chimaera, Black-Figure Kylix with Bellerophon Fighting the Chimaera, Bellerophon, Pegasus, Boreads Painter, public health

**Figure:**
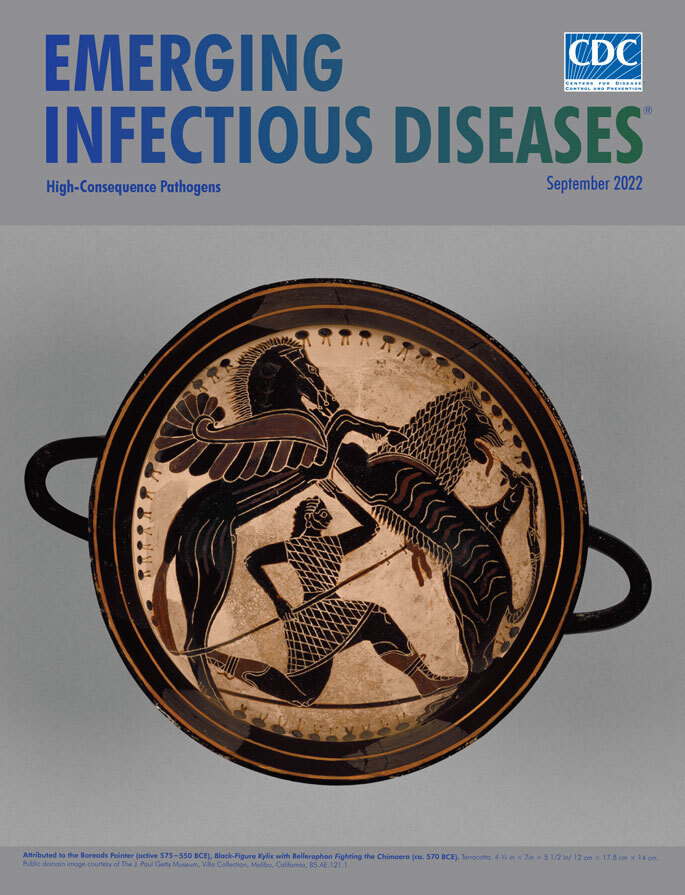
**Attributed to the Boreads Painter (active 575−550 bce).** Black-Figure Kylix with Bellerophon Fighting the Chimaera (ca. 570 bce). Terracotta. 4¾ in × 7 in × 5½ in/12 cm × 17.8 cm × 14 cm. Public domain image courtesy of the J. Paul Getty Museum, Villa Collection, Malibu, California, USA, 85.AE.121.1.

Disease outbreaks caused by high-consequence infectious pathogens can disrupt human lives in myriad ways by causing health, societal, and economic problems that require nimble and creative strategic responses. Similar disruptions can result from mythical monsters running roughshod over imaginary kingdoms. 

The Chimaera, originally from Greek mythology, was such a monster. This hybrid creature terrorized the mythical countryside, killing humans and livestock, creating fear and panic. The earliest known description of the Chimaera comes from the 8th century bce epic poem *The Iliad*, in which the Greek poet Homer described it as “a thing of immortal make, not human, lion fronted and snake behind, a goat in the middle, and snorting out the breath of the terrible flame of bright fire.”

Art historian Marilyn Low Schmitt wrote, “The story of Bellerophon and the Chimaera was one of the earliest legends to be represented in Greek art. In the legend Bellerophon traveled from Argos to Tiryns to Lycia, where the Lycian king, Iobates, commanded him to slay the Chimaera.” Bellerophon, a young Corinthian warrior who was a son of the Greek god Poseidon, confronted an intimidating challenge in being pitted against this deadly monster. But there was also an element of subterfuge behind King Iobates’ motives beyond any desire to rid the land of this scourge.

Iobates’ daughter Stheneboea was married to the neighboring king, Proteus. She had falsely accused Bellerophon of sexual impropriety. Unbeknownst to Proteus, Bellerophon had actually snubbed Stheneboea’s advances. But believing he had been dishonored, Proteus secretly asked his father-in-law, Iobates, for his complicity in eliminating the warrior whom Proteus dared not challenge directly. Both kings no doubt assumed the Chimaera would slay Bellerophon. 

Indeed, had Bellerophon not tamed the flying horse Pegasus, gifted to him by the gods to aid in this endeavor, he would most likely have suffered a grim fate. Mounted on Pegasus, Bellerophon flew above the Chimaera, where he was able to surveil the situation, rather than lunging headfirst into battle. From this vantage point, Bellerophon repeatedly shot arrows into the enraged monster but could not kill it. He delivered the coup de grâce, when, as Schmitt noted, he connived “to drop into the Chimaera’s throat a lump of lead which would melt in the fiery breath and sear its organs.” How Bellerophon acquired a lump of lead is not revealed, and in other versions of this tale, Bellerophon prevailed by lodging a lead-tipped spear point in the creature’s throat, where it melted. 

This month’s cover image shows the interior of a kylix, a broad-bowled drinking cup, emblazoned with Bellerophon and Pegasus battling the Chimaera. Art historians have attributed many similarly styled works of pottery from this era in Greek history to an artisan known as the Boreads Painter. Although the identity of the Boreads Painter remains unknown, the British Museum observes that “Nevertheless consistent individual characteristics of style suggest the existence of a unique artistic personality.” This anonymous artisan, as the British Museum documents, has been given the name of the Boreads Painter for an image he created of the sons of Boreas, Greek god of the North Wind. That image appears on what the British Museum describes as “his masterpiece, a cup in the Villa Giulia in Rome decorated on the tondo with an image of a pair of Boreads pursuing a pair of Harpies.” 

On the basis of the volume of similarly styled artifacts, historians believe that this artist operated one of the more important and productive pottery studios in Sparta from 575 to 550 bce. Some of his distinctive stylistic elements include his specific way of drawing eyes, ears, knees, and hips and his practice of encircling images with a band of pomegranates.

The J. Paul Getty Museum, which houses this kylix, describes the cup’s interior in this manner: “In his right hand, the hero holds the reins of his winged horse, Pegasus, who rears up to meet the monster. Bellerophon is shown in the kneeling pose used to characterize quick movement in Greek art of the Archaic period (about 700–480 B.C.).” Although this depiction of the battle differs from written descriptions of Bellerophon attacking while mounted on Pegasus, the Getty Museum offers a practical reason for such a departure: “The unique, symmetrical arrangement of the rearing horse and monster framing the hero is the result of the artist's attempt to find creative ways to fill the circular area of the interior of a cup.”

Bellerophon’s success was predicated on having a reliable ally in Pegasus and from his strategic planning literally on the fly. Those on the front lines of public health efforts to prevent, investigate, and mitigate outbreaks of diseases caused by high-consequence pathogens are also engaged in a high-stakes endeavor. Monkeypox, rabies, Nipah virus infection, and a cluster of hemorrhagic fever diseases (e.g., Ebola virus disease, Marburg hemorrhagic fever, Crimean-Congo hemorrhagic fever, and Rift Valley fever) are among the diseases caused by high-consequence pathogens. Many pathogens are themselves “chimeric,” because organisms can combine genetic material to mutate into variants that create challenges for treatment and control, requiring innovative and agile thinking, as demonstrated by Bellerophon while slaying the Chimaera.

## References

[1] Belay ED, Monroe SS. Low-incidence, high-consequence pathogens. Emerg Infect Dis. 2014;20:319–21. 10.3201/eid2002.131748 24596949 PMC3901478

[2] British Museum. The Boreads Painter [cited 2022 Jun 9]. https://www.britishmuseum.org/collection/term/BIOG57451

[3] Cartwright M. Bellerophon [cited 2022 Jul 23]. https://www.worldhistory.org/Bellerophon

[4] Centers for Disease Control and Prevention. Division of High Consequence Pathogens and Pathology [cited 2022 Jul 18]. https://www.cdc.gov/ncezid/dhcpp/index.html

[5] Getty Museum. Black-figure kylix [cited 2022 Jul 14]. https://www.getty.edu/art/collection/object/108DSR

[6] Getty Museum. Lakonian black-figure kylix; detached fragments [cited 2022 Jul 14]. https://www.getty.edu/art/collection/object/103VPC

[7] Homer. The Iliad. Book VI (179−182). Lattimore R, translator. Chicago: The University of Chicago Press; 1951, 2011.

[8] Schmitt ML. Bellerophon and the chimaera in archaic Greek art. Am J Archaeol. 1966;70:341–7. 10.2307/502324

